# Sterile α-motif domain requirement for cellular signaling and survival

**DOI:** 10.1074/jbc.RA119.011895

**Published:** 2020-04-02

**Authors:** Suhita Ray, Linda Chee, Daniel R. Matson, Nick Y. Palermo, Emery H. Bresnick, Kyle J. Hewitt

**Affiliations:** ‡ Department of Genetics, Cell Biology and Anatomy, Fred and Pamela Buffett Cancer Center, University of Nebraska Medical Center, Omaha, Nebraska 68198; § University of Wisconsin–Madison Blood Research Program, Department of Cell and Regenerative Biology, Carbone Cancer Center, University of Wisconsin School of Medicine and Public Health, Madison, Wisconsin 53705; ¶ Holland Computing Center, University of Nebraska-Lincoln, Lincoln, Nebraska 68588

**Keywords:** protein motif, erythropoiesis, stem cell factor (SCF), stress, structure–function, hemolytic anemia, proto-oncogene c-Kit (c-Kit), receptor tyrosine kinase, sterile α-motif, sterile α-motif domain containing 14 (SAMD14)

## Abstract

Hundreds of sterile α-motif (SAM) domains have predicted structural similarities and are reported to bind proteins, lipids, or RNAs. However, the majority of these domains have not been analyzed functionally. Previously, we demonstrated that a SAM domain-containing protein, SAMD14, promotes SCF/proto-oncogene c-Kit (c-Kit) signaling, erythroid progenitor function, and erythrocyte regeneration. Deletion of a Samd14 enhancer (Samd14–Enh), occupied by GATA2 and SCL/TAL1 transcription factors, reduces SAMD14 expression in bone marrow and spleen and is lethal in a hemolytic anemia mouse model. To rigorously establish whether Samd14–Enh deletion reduces anemia-dependent c-Kit signaling by lowering SAMD14 levels, we developed a genetic rescue assay in murine Samd14–Enh^−/−^ primary erythroid precursor cells. SAMD14 expression at endogenous levels rescued c-Kit signaling. The conserved SAM domain was required for SAMD14 to increase colony-forming activity, c-Kit signaling, and progenitor survival. To elucidate the molecular determinants of SAM domain function in SAMD14, we substituted its SAM domain with distinct SAM domains predicted to be structurally similar. The chimeras were less effective than SAMD14 itself in rescuing signaling, survival, and colony-forming activities. Thus, the SAMD14 SAM domain has attributes that are distinct from other SAM domains and underlie SAMD14 function as a regulator of cellular signaling and erythrocyte regeneration.

## Introduction

Erythrocyte developmental mechanisms can differ during embryogenesis, adult homeostasis, and regenerative responses to injury or stress (1, 2). In anemia, the increased erythropoiesis demand is met by augmented erythroid progenitor activity in the bone marrow or at extramedullary sites (3–6). A cell population termed “stress erythroid progenitors” responds to diverse anemia-dependent paracrine signals, including erythropoietin (Epo)^2^ and stem cell factor (SCF), to induce rapid expansion and differentiation, thereby re-establishing homeostasis (7). In severe hemolytic anemia, expression of sterile α-motif (SAM) domain protein-14 (Samd14) increases in spleen erythroid progenitors, enhances SCF-mediated cell signaling via the receptor tyrosine kinase c-Kit, and is required for survival of mice (4). c-Kit signaling promotes proliferation, differentiation, survival, and/or migration (8–11). SCF activation of c-Kit supports erythroid precursor proliferation via parallel MAPK and PI3K/Akt-signaling pathways (12–14).

Anemia-dependent transcriptional induction of Samd14 expression requires GATA-binding transcription factors. To establish whether the GATA2-occupied Samd14-enhancer (Samd14–Enh) is essential *in vivo*, we generated a mutant mouse strain lacking this site (4). Samd14–Enh has attributes resembling the *Gata2* +9.5 intronic enhancer, an essential trigger of HSC emergence in the mouse embryo (15–17) and regulator of progenitor cell fate (18). Despite the common attributes, steady-state hematopoiesis is normal in Samd14–Enh^−/−^ mice (4). By contrast, Samd14–Enh promotes erythrocyte regeneration and survival of mice in severe anemia. Anemia induces *Samd14* expression, and Samd14–Enh mediates the transcriptional activation (4). Beyond Samd14–Enh, we identified a cohort of additional +9.5-like enhancers harboring GATA2-occupied E-box–GATA elements, analogous to Samd14–Enh, which are predicted to have important functions to control hematopoiesis and/or stress erythroid progenitors (19, 20). The cell-type specificity of Samd14–Enh function is illustrated by its importance for *Samd14* transcription in bone marrow and splenic myeloerythroid progenitors, but not in brain. As Samd14–Enh promotes erythrocyte regeneration, these findings extend the function of E-box–GATA enhancer elements beyond developmental and steady-state hematopoiesis (15, 20, 21).

Samd14 contains a C-terminal SAM domain of unknown function and importance. SAM domains are common in the mammalian proteome and vary in size from ∼65 to 70 amino acids, which fold into five short α-helical regions (22, 23). Despite predictions of shared structural features, SAM domains have been implicated in diverse biochemical functions, including mediating protein self-association and heterologous interactions with other proteins (24–26). SAM domain proteins can regulate important physiological processes, *e.g.* SAMHD1 controls auto-immunity (27). In addition, SAM domain proteins can be implicated in human pathogenic mechanisms, *e.g.* SAMD9 and SAMD9L mutations are linked to bone marrow failure that can progress to myeloid malignancies (28, 29). No general principles have emerged to explain SAM domain function, and whether individual SAM domains can substitute for others is unknown. Thus, we devised a genetic complementation system with Samd14 and mutant chimeric proteins in which the Samd14 SAM domain was replaced with SAM domains from other SAM domain proteins. Guided by insights derived from structural prediction tools, we conducted structure/function analyses. Our results demonstrate that the Samd14 SAM domain promotes c-Kit–mediated cellular signaling to regulate progenitor function, and this SAM domain has functional attributes unique from those of structurally-related SAM domains.

## Results

### Samd14 genetic rescue assay in Samd14 enhancer–mutant progenitor cells

**Figure 1.**
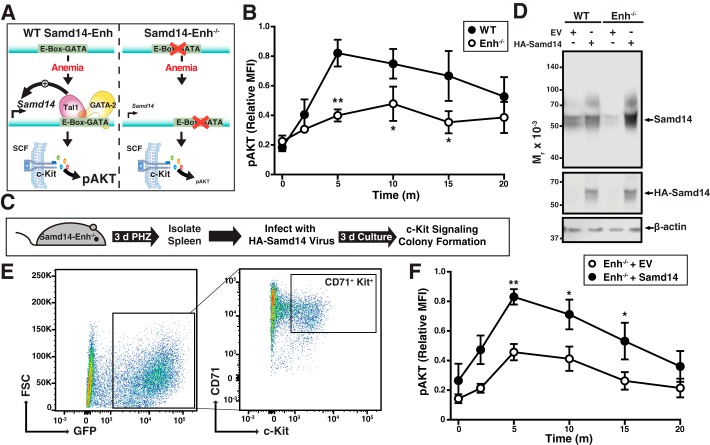
**Samd14 genetic complementation in Samd14–Enh^−/−^ erythroid progenitor cells rescues SCF/c-Kit signaling.** *A,* model depicting the Samd14–Enh requirement for induction of Samd14 expression upon anemia and Samd14-mediated promotion of c-Kit signaling (phosphorylation of AKT). In Samd14–Enh-deleted (Enh^−/−^) cells, pAKT levels are lower in response to SCF (4). *B,* phospho-flow cytometry quantitating pAKT levels in WT (*n* = 6) and Enh^−/−^ (*n* = 6) over a 20-min (*m*) time course of SCF stimulation. *C,* experimental layout of spleen *ex vivo* retroviral infection and culture. *D,* representative Western blotting of WT and Enh^−/−^ protein lysates infected with EV or full-length HA-tagged Samd14. *E,* flow cytometric analysis depicting the population of GFP^+^CD71^+^Kit^+^ cultured spleen erythroid progenitors. *F,* phospho-flow cytometry quantitating pAKT levels in infected WT (*n* = 6) and Enh^−/−^ (*n* = 6) over a 20-min time course of SCF stimulation. *Error bars* represent the standard error of the mean (S.E.). *, *p* < 0.05; **, *p* < 0.01 (two-tailed unpaired Student's *t* test). *FSC*, forward scatter.

A composite E-box–GATA *cis*-element confers Samd14 expression in spleen and bone marrow hematopoietic cells (4, 19). Loss of the Samd14-enhancer (Samd14–Enh) prevented anemia-induced Samd14 expression in spleen and attenuated c-Kit signaling in erythroid progenitors (Fig. 1*A*). Thus, Samd14–Enh-mediated up-regulation of *Samd14* expression promotes c-Kit signaling in splenic progenitors to support erythrocyte regeneration during anemia. *Ex vivo*, Samd14–Enh^−/−^ erythroid progenitors (CD71^+^Ter119^−^Kit^+^) exhibited 2.1- and 1.6-fold lower phospho (serine 473) AKT (pAKT) *versus* WT in response to 5 or 10 min of SCF treatment, respectively (Fig. 1*B*).

To determine whether reduced c-Kit signaling resulting from Samd14 down-regulation is reversible, we developed a genetic complementation assay involving restoration of Samd14 expression in Samd14–Enh^−/−^ splenic progenitors isolated from anemic mice (Fig. 1*C*). Lin^−^ progenitors were isolated from the spleens of mice treated with the erythrocyte-lysing and anemia-inducing chemical phenylhydrazine (PHZ) for 3 days. Retroviral expression of HA-tagged Samd14 (HA–Samd14) increased Samd14 protein levels relative to empty vector (EV) controls (Fig. 1*D*). Consistent with prior studies (4), Samd14 protein appears as a diffuse band by SDS-PAGE. After 72 h under culture conditions that favor erythroid precursor proliferation, cultures were treated with SCF for up to 20 min, and a phospho-flow cytometry assay was used to quantify signaling pathway activation in infected GFP^+^CD71^+^Kit^+^ cells (Fig. 1*E*). After 5 min of SCF treatment, pAKT levels were 1.8-fold higher (*p* = 0.003) in Samd14–Enh^−/−^ cells expressing HA–Samd14 compared with EV controls (Fig. 1*F* and Fig. S1). As the SCF/c-Kit signaling defect in Samd14–Enh^−/−^ splenic progenitors was rescued by restoring physiological levels of Samd14, the defect results from insufficient Samd14 expression.

**Figure 2.**
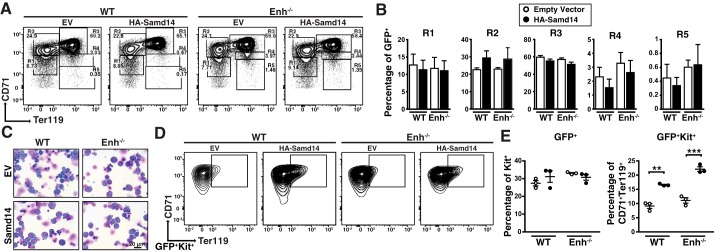
**Samd14 genetic complementation in Samd14–Enh^−/−^ erythroid progenitor cells increases CD71^+^Ter119^+^Kit^+^ cells without impacting erythroid maturation.** *A,* representative flow cytometric analysis of CD71 and Ter119 cells following retroviral infection of EV or Samd14 in WT and Samd14–Enh^−/−^ (*Enh*^−/−^) spleen cultures. *R1–R5* represent progressive stages of erythroid maturation. *B,* quantitation of infected R1–R5 cells in WT and Samd14–Enh^−/−^ cells. *C,* representative Wright-Giemsa–stained cells in erythroid differentiation conditions. ×40 magnification; *scale bar* = 20 μm. *D,* representative flow cytometric analysis of CD71 and Ter119 staining after gating for GFP^+^Kit^+^ cells. *E, left*, quantitation of infected c-Kit^+^ cells. *Right,* quantitation of CD71^+^Ter119^+^ cells within infected c-Kit^+^ population. *Error bars* represent the standard error of the mean (S.E.). **, *p* < 0.01; ***, *p* < 0.001 (two-tailed unpaired Student's *t* test).

Erythroid differentiation of lineage-depleted splenic progenitors from PHZ-treated animals was monitored using CD71 and Ter119 surface markers. The percentage of infected cells in each experiment is shown in Table S1. Early erythroid precursors reside in R1 (CD71^low^, Ter119^−^) and R2 (CD71^high^, Ter119^−^) compartments, with progressively differentiating erythroblasts in R3 (CD71^high^, Ter119^+^), R4 (CD71^med^, Ter119^+^), and R5 (CD71^low^, Ter119^+^) (Fig. 2*A*). When quantitated, there were no differences in cellularity between WT and Samd14–Enh^−/−^ cells after 3 days of culture (Fig. 2*B*). Giemsa staining confirmed the similar morphologies of EV- and Samd14-infected cells (Fig. 2*C*). However, Samd14 expression increased the percentage of CD71^+^Ter119^+^c-Kit^+^ cells relative to EV cells (Fig. 2*D*). Whereas the overall percentage of c-Kit^+^ cells in the HA–Samd14-infected cell population did not change, CD71^+^Ter119^+^ cells within the c-Kit^+^ population increased 2.0 ± 0.1-fold (*p* = 0.002) (Fig. 2*E*). Thus, restoring Samd14 expression in Samd14–Enh^−/−^ spleen erythroid progenitors increased the percentage of CD71^+^Ter119^+^Kit^+^ cells without impacting overall erythroid maturation.

### SAM domain-dependent cellular survival mechanism

**Figure 3.**
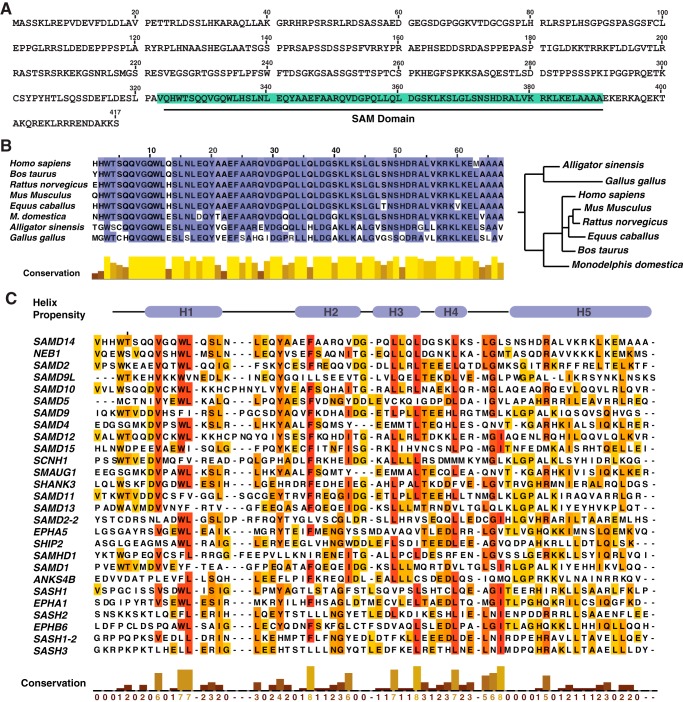
**Samd14 SAM is evolutionarily conserved.** *A,* sequence of murine Samd14. *B, left,* sequence alignment of the SAM domain of Samd14 among vertebrate species. Alignment was conducted with Clustal Omega and visualized with JalView. *Right,* phylogenetic tree, generated by neighbor-joining of aligned sequence (JalView) of Samd14 among organisms that contain the annotated gene. *C,* sequence alignment of SAM domains. *H1–H5* represent helical regions determined by *in silico* alignment of Samd14 with the solved structure of SHIP2 (37).

Samd14 is predicted to harbor a SAM domain from amino acid residues 323–390 of unknown function (Fig. 3*A*). Human SAMD14 shares 94% protein identity with murine Samd14, and both proteins contain a 68-amino acid C-terminal SAM domain. The Samd14 SAM domain is evolutionarily conserved in many vertebrate species, with only three residues varying between humans and mice (Fig. 3*B*). SAM domains can mediate protein self-association, interaction with other proteins, or lipids or RNA binding (24, 25). However, principles governing SAM domain function are not established, and one cannot predict whether a given SAM domain is essential, modulatory, or functionally insignificant. SAM domain protein families have been implicated in signal transduction (19, 30, 31), transcription (32), translation (33), and protein transport (34).

**Figure 4.**
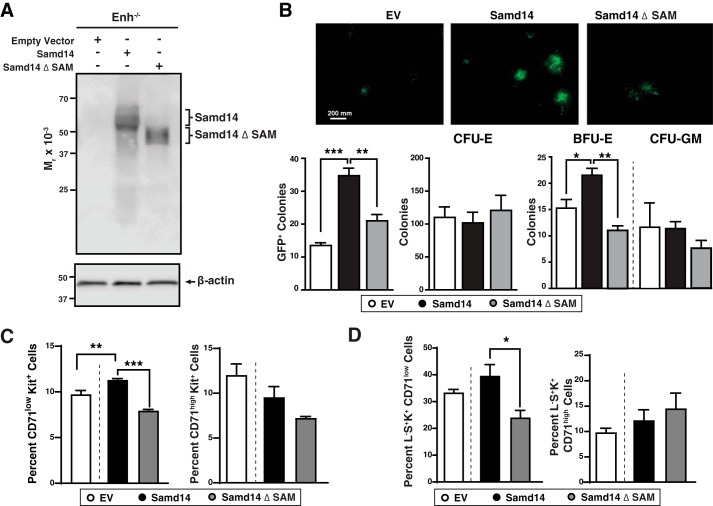
**Samd14 SAM domain facilitates Samd14-dependent progenitor activity.** *A,* Samd14 Western blotting of spleen progenitors retrovirally-infected with EV, HA-tagged Samd14, and a SAM domain-deleted mutant of Samd14 (*Samd14* Δ*SAM*). *B, top,* representative images of GFP^+^ colonies (*n* = 4 in each condition). *Scale bar* = 200 mm. *Bottom,* quantitation of GFP^+^ colonies, CFU-E, BFU-E, and CFU-GM (*n* = 4). *C, left,* quantitation of the percent of retrovirally-infected CD71^low^Kit^+^ (*n* = 3 in each condition). *Right,* quantitation of the percent of retrovirally-infected CD71^high^Kit^+^ (*n* = 3 in each condition). *D, left,* quantitation of the percent of retrovirally-infected Lineage^−^ Sca1^+^Kit^+^CD71^low^ (*L*^−^*S*^+^*K*^+^*CD71^low^*) (*n* = 3 in each condition). *Right*, quantitation of the percent of retrovirally-infected Lineage^−^ Sca1^+^Kit^+^CD71^high^ (*L*^−^*S*^+^*K*^+^*CD71^high^*). *Error bars* represent the standard error of the mean (S.E.). *, *p* < 0.05; **, *p* < 0.01; ***, *p* < 0.001 (two-tailed unpaired Student's *t* test).

To identify potentially important structural features of the Samd14 SAM domain, we utilized *in silico* homology modeling using known NMR-derived protein structures. Samd14–SAM was predicted to have five α-helices that align with other SAM proteins (35) (Fig. 3*C*). Sequence similarity of the Samd14 SAM domain with other SAM domains varied from 57% identity with Neurabin-1 (Neb1) SAM to 20% with SHIP-2 SAM. To determine whether the Samd14 SAM domain regulates progenitor function, we generated a SAM domain–deleted construct of Samd14 (Samd14 ΔSAM) and analyzed its function in the genetic rescue assay with cells from Samd14–Enh-deleted bone marrow and spleen (Fig. 4*A*). In CFU assays, Samd14 increased GFP^+^ colony formation 2.7-fold (*p* = 0.004). By contrast, Samd14 ΔSAM did not significantly affect colonies compared with EV (Fig. 4*B*). Relative to Samd14, Samd14 ΔSAM induced 1.9-fold fewer (*p* = 0.0006) burst-forming unit–erythroid (BFU-E) colonies (Fig. 4*B*). Samd14 or Samd14 ΔSAM expression did not impact the levels of CFU-erythroid (CFU-E) and CFU-granulocyte/monocyte (CFU-GM) colonies (Fig. 4*B*). As an indirect measure of progenitor levels, we utilized two flow-cytometry approaches to identify populations of cells enriched in BFU-E or CFU-E (6, 36). Samd14 expression increased the number of CD71^low^Kit^+^ cells that are enriched in BFU-E. Samd14 ΔSAM has 1.4-fold lower CD71^low^Kit^+^ than Samd14 (*p* < 0.0001) (Fig. 4*C*). The levels of CD71^high^Kit^+^ cells, enriched in CFU-E, were not altered between Samd14 and Samd14 ΔSAM. The levels of Lin^−^Sca1^+^Kit^+^CD71^low^ is 1.65-fold lower in Samd14 ΔSAM *versus* Samd14 (*p* = 0.036) (Fig. 4*D*). Thus, the Samd14 SAM domain is an important determinant of progenitor cell levels and function.

**Figure 5.**
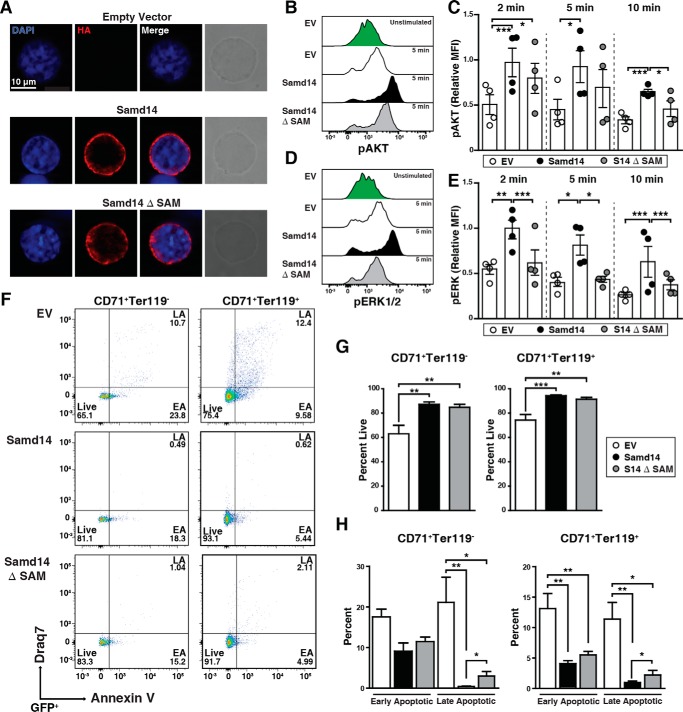
**Samd14 SAM domain increases MAPK signaling and cellular survival.** *A,* representative immunofluorescence images of retrovirally-infected spleen erythroid progenitors stained with rabbit anti-HA antibody and Alexa Fluor 594 (*red*) and DAPI (*blue*). Phase-contrast images indicate cell size. ×100 magnification; *scale bar* = 10 μm. *B,* overlaid histograms of pAKT fluorescence intensity in GFP^+^c-Kit^+^CD71^+^ spleen erythroid progenitors at 5 min post-SCF stimulation. *C,* quantitation of pAKT median fluorescence intensity at 2, 5, and 10 min post-stimulation with SCF (10 ng/ml) (*n* = 4 in each condition). *D,* overlaid histograms of pERK1/2 fluorescence intensity in GFP^+^c-Kit^+^CD71^+^ spleen erythroid progenitors at 5 min post-SCF stimulation. *E,* quantitation of pERK1/2 median fluorescence intensity at 2, 5, and 10 min post-stimulation with SCF (10 ng/ml) (*n* = 4 in each condition). *F,* representative flow cytometric analysis of membrane-impermeable DNA dye (*Draq7*) and anti-annexin V Pacific Blue (AnnV). Cells were first segregated based on GFP, CD71, and Ter119. Draq7^−^ AnnV^−^ = live; Draq7^−^AnnV^+^ = early apoptotic (*EA*), and Draq7^+^AnnV^+^ = late apoptotic (*LA*). *G, left,* quantitation of percent live cells in CD71^+^Ter119^−^ population (*n* = 9). *Right,* quantitation of percent live cells in CD71^+^Ter119^+^ population (*n* = 9). *H, left,* quantitation of early and late apoptotic cells in CD71^+^Ter119^−^ population (*n* = 9). *Right,* quantitation of early and late apoptotic cells in CD71^+^Ter119^+^ population (*n* = 9). *Error bars* represent the standard error of the mean (S.E.). *, *p* < 0.05; **, *p* < 0.01; ***, *p* < 0.001 (two-tailed unpaired Student's *t* test).

To distinguish among mechanisms of Samd14 SAM domain activity, we tested whether Samd14 ΔSAM subcellular localization resembles or contrasts from that of Samd14. Immunofluorescence analysis with cultures of splenic erythroid progenitors revealed both Samd14 and Samd14 ΔSAM localized throughout the cytoplasm (Fig. 5*A*). To determine whether Samd14 co-localized with c-Kit, we conducted immunofluorescence using structured illumination microscopy followed by quantitative co-localization analysis (Fig. S2*A*). In cells expressing either Samd14 or Samd14 ΔSAM, Samd14 and c-Kit localization patterns did not correlate (*r* < 0.1), suggesting that the majority of Samd14 and c-Kit do not co-localize (Fig. S2*B*). To test whether Samd14 co-localizes with c-Kit after c-Kit activation, we treated cells with SCF for 5 min prior to immunofluorescence analysis. Whereas the correlation values were low in both SCF-treated *versus* nontreated cells, the correlation coefficient between Samd14 or Samd14 ΔSAM in relation to c-Kit increased 3.2- and 2.6-fold, respectively (Fig. S2).

We tested whether Samd14 SAM is important for Samd14 promotion of c-Kit signaling. Samd14- and Samd14 ΔSAM-infected cells were treated with SCF for various times and fixed prior to phospho-flow cytometric analysis. GFP^+^Kit^+^ cells were analyzed after a 72-h culture (Fig. S3). SCF treatment of Samd14- (but not Samd14 ΔSAM)-expressing cells for 5 min increased pAKT compared with EV controls (Fig. 5*B*). At 10 min post-SCF treatment, Samd14 expression increased pAKT levels 1.6-fold (*p* = 0.0065); Samd14 ΔSAM did not alter pAKT levels (Fig. 5*C*). Expression of Samd14, but not Samd14 ΔSAM, increased pERK levels relative to EV controls (Fig. 5*D*). In Samd14-expressing cells, pERK increased 2.0-fold (*p* = 0.003) at 2 min, 2.0-fold (*p* = 0.016) at 5 min, and 2.5-fold (*p* = 0.002) at 10 min *versus* EV control. In Samd14 ΔSAM-expressing cells, pERK levels, relative to EV controls, were unaffected and significantly lower than in Samd14-expressing cells (Fig. 5*E*). In bone marrow progenitor cultures, Samd14 or Samd14 ΔSAM expression did not alter SCF-mediated induction of pAKT (Fig. S4). Thus, in spleen erythroid progenitors, the Samd14 SAM domain promoted c-Kit signaling via both MAPK and PI3K/Akt pathways.

Under conditions of hemolytic anemia, Samd14–Enh confers cell survival in erythroid splenic progenitors (4). To test the role of Samd14 SAM in Samd14-mediated cellular survival, we utilized a Samd14–Enh^−/−^ spleen erythroid progenitor culture system and quantified the percentage of live (annexinV^−^Draq7^−^), early apoptotic (EA; annexinV^+^Draq7^−^), and late apoptotic cells (LA; annexinV^+^Draq7^+^) cells (Fig. 5*F*). Whereas 63% of GFP^+^CD71^+^Ter119^+^ control cells were live cells, expressing Samd14 and Samd14 ΔSAM increased the percentage of live cells to 87% (*p* = 0.004) and 84% (*p* = 0.009), respectively (Fig. 5*G*). In GFP^+^CD71^+^Ter119^−^ cells, Samd14 expression did not alter the percentage of early apoptotic cells. However, the percent of late apoptotic cells was considerably lower in Samd14-expressing *versus* control cells (0.43 *versus* 21%) (Fig. 5*H*, *left*). Samd14 ΔSAM-expressing CD71^+^Ter119^−^ cultures contained a 2.8-fold (*p* = 0.039) higher percentage of late apoptotic cells. Commensurate with increased viability, Samd14 expression decreased the percentage of GFP^+^CD71^+^Ter119^+^ early and late apoptotic cells *versus* control by 3.2-fold (*p* = 0.002) and 12-fold (*p* = 0.002) respectively (Fig. 5*H*, *right*). Compared with Samd14, Samd14 ΔSAM-expressing CD71^+^Ter119^+^ cells contained a 3.2-fold (*p* = 0.043) higher percentage of late apoptotic cells (Fig. 5*H*). Within the c-Kit–positive population of GFP^+^CD71^+^Ter119^−^ cells, Samd14 expression decreased the percentage of late apoptotic cells *versus* control by 33-fold (*p* = 0.008) (Fig. 5*A*). Compared with Samd14, Samd14 ΔSAM-expressing CD71^+^Ter119^+^ cells contained a 5.8-fold (*p* = 0.016) higher percentage of late apoptotic cells (Fig. S5*B*). The Samd14 SAM domain therefore promotes Samd14 function in cellular signaling and survival.

### Elucidating SAM domain function using chimeric SAM domain proteins

**Figure 6.**
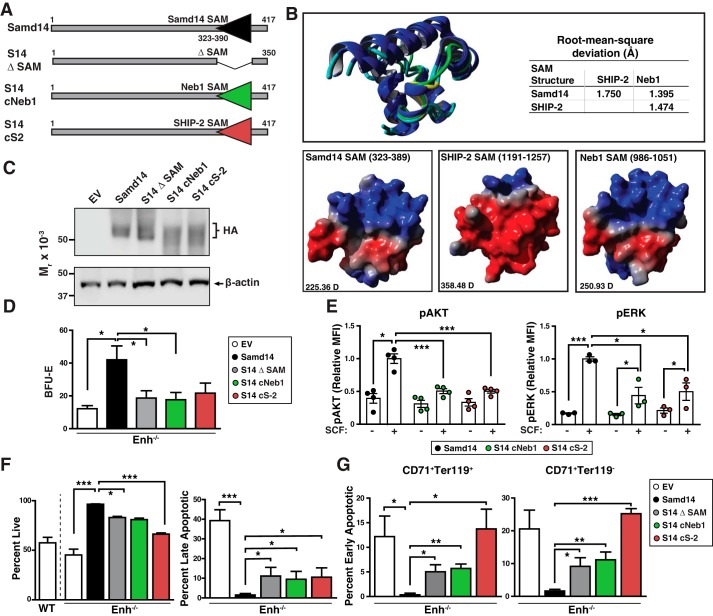
**Dissecting Samd14 SAM domain function with chimeric SAM domain proteins.** *A,* schematic representation of fusion constructs. *B, top,* overlay of predicted SAM domains of Samd14 and Neb1 with SHIP-2. *Blue,* α helix; *teal,* coil; *green,* bend; and *yellow,* turn. *Bottom,* surface models of Samd14, SHIP-2, and Neb1 SAM domains. *Red*, negatively charged area; *blue*, positive; *gray,* neutral. *D*, dipole/debye. *C,* Western blotting of spleen progenitors retrovirally-infected with EV, HA-tagged Samd14, a SAM domain-deleted mutant of Samd14 (*Samd14* Δ*SAM*), and Samd14 with SAM domain sequence of Neurabin-1 (*S14–cNeb1*) and Samd14 with SAM domain sequence of SHIP-2 (*S14-cS2*). *D,* quantitation of BFU-E colonies from 12-h cultured GFP^+^ cells (*n* = 7, BFU-E numbers normalized to colonies per 3 × 10^4^ GFP^+^ cells). *E,* quantitation of pAKT and pERK1/2 median fluorescence intensity post-stimulation with SCF (10 ng/ml). (*n* = 4 for each condition). *F,* quantitation of flow cytometric analysis of noncell membrane permeating DNA dye (Draq7) and anti-annexin V Pacific Blue (AnnV). Cells were first segregated based on GFP^+^, CD71^+^, and Ter119^+^. Draq7^−^AnnV^−^, live; and Draq7^+^AnnV^+^, late apoptotic. *G,* quantitation of flow cytometric analysis of noncell membrane permeating DNA dye (Draq7) and anti-annexin V Pacific Blue (AnnV). *Left,* cells were segregated based on GFP^+^, CD71^+^, and Ter119^+^. *Right,* cells were segregated based on GFP^+^, CD71^+^, and Ter119^−^. Draq7^−^AnnV^+^, early apoptotic (*n* = 3). *Error bars* represent the standard error of the mean (S.E.). *, *p* < 0.05; **, *p* < 0.01; ***, *p* < 0.001 (two-tailed unpaired Student's *t* test).

To identify SAM domain molecular attributes that contribute to Samd14-dependent SCF/c-Kit signaling and cellular survival, we generated chimeric Samd14 proteins by replacing the 68-amino acid SAM domain sequence between residues 323 and 390 of Samd14 with analogous SAM domains from other proteins. Samd14–SAM was replaced by the 68-amino acid SAM domain sequence of Neurabin-1 (residues 986–1053), highly homologous to Samd14 (S14–cNeb1), or the 68-amino acid SHIP-2 SAM (residues 1190–1257) (S14-cS2) (Fig. 6*A*). As the NMR structure of the SHIP-2 SAM domain is solved (37), we used homology modeling to generate atomic resolution in *in silico* projections of the Samd14 and Neb1 SAM domains. The folded SAM domains are predicted to be structurally similar (RMSD <2.0 Å) (Fig. 6*B*). No salt bridges or hydrogen bonding appears to exist between the helices, suggesting that the fold is stabilized by weakly polar interactions within the hydrophobic core. Despite the high congruence of the folded structure, the calculated dipole (D) moments for Samd14 SAM (225.36 D), Neb1 SAM (250.93 D), and SHIP-2 SAM (358.48 D) suggest that each SAM domain exhibits distinct electrostatic surface potentials (Fig. 6*B*).

As Samd14 SAM enhances Samd14 function in stress erythroid progenitors, we tested whether the Neb1 or SHIP-2 SAM domains could reconstitute activity of Samd14 lacking its SAM domain. SAM domain chimeric proteins were expressed in spleen erythroid progenitors isolated from Samd14–Enh^−/−^ mice using our genetic rescue assay (Fig. 6*C*). Similar to Samd14, S14–cNeb1 and S14-cS2 proteins localized within the cytoplasm of spleen erythroid progenitors (Fig. S6). Neither S14–cNeb1 nor S14-cS2 increased BFU-E numbers relative to EV controls (Fig. 6*D*). Compared with Samd14, S14-Neb1- or S14-cS2-expressing stress progenitors exhibited 2.4-fold (*p* = 0.03) and 1.9-fold (*p* = 0.05) colonies, respectively (Fig. 6*D*). S14-cS2 and S14–cNeb1 have 1.2-fold lower CD71^low^Kit^+^ than Samd14 (*p* = 0.014 and *p* = 0.043), respectively (Fig. S7). Cell signaling analyses were conducted to test whether S14–cNeb1 or S14-cS2 are competent to facilitate SCF/c-Kit signaling in primary stress erythroid progenitors. In S14–cNeb1- or S14-cS2–expressing cells, SCF-mediated AKT and ERK activation was lower than in cells expressing Samd14 (Fig. 6*E*). Relative to Samd14-expressing cells, S14–cNeb1 and S14-cS2 exhibited 2.3-fold (*p* = 0.01) and 2.0-fold (*p* = 0.02) lower pERK in response to SCF, respectively (Fig. 6*E*). To test the capacity of SAM domain fusion proteins to confer cellular survival, we quantified the percentage of live (annexinV^−^Draq7^−^), EA (annexinV^+^Draq7^−^), and LA (annexinV^+^Draq7^+^) CD71^+^Ter119^−^ cells (Fig. 6*F*). In S14–cNeb1- and S14-cS2–expressing cells, the percentage of live GFP^+^CD71^+^Ter119^−^ cells decreased from 96% in controls to 81% (*p* = 0.006) and 66% (*p* = 0.001), respectively. An increased percentage of late apoptotic cells was detected in S14–cNeb1 (9.5%) and S14-cS2 (10.5%), relative to Samd14 (1.5%) (Fig. 6*F*). Among GFP^+^CD71^+^Ter119^+^, the percentage of late apoptotic cells in S14–cNeb1 (5.7%) and S14-cS2 (13.7%) was 17-fold (*p* = 0.005) and 40-fold (*p* = 0.03) higher than Samd14 (0.3%) (Fig. 6*G*, *left*). Within the less-differentiated GFP^+^CD71^+^Ter119^−^ cells, S14–cNeb1 (11.7%) and S14-cS2 (22.4%) contained 23-fold (*p* = 0.001) and 45-fold (*p* = 0.0005) more early apoptotic cells compared with Samd14 (0.5%) (Fig. 6*G*, *right*). As the SHIP-2 and Neb1 SAM domains did not reconstitute Samd14 function, the Samd14 SAM domain has unique molecular determinants that endow Samd14 with the capacity to regulate cellular signaling and survival.

**Figure 7.**
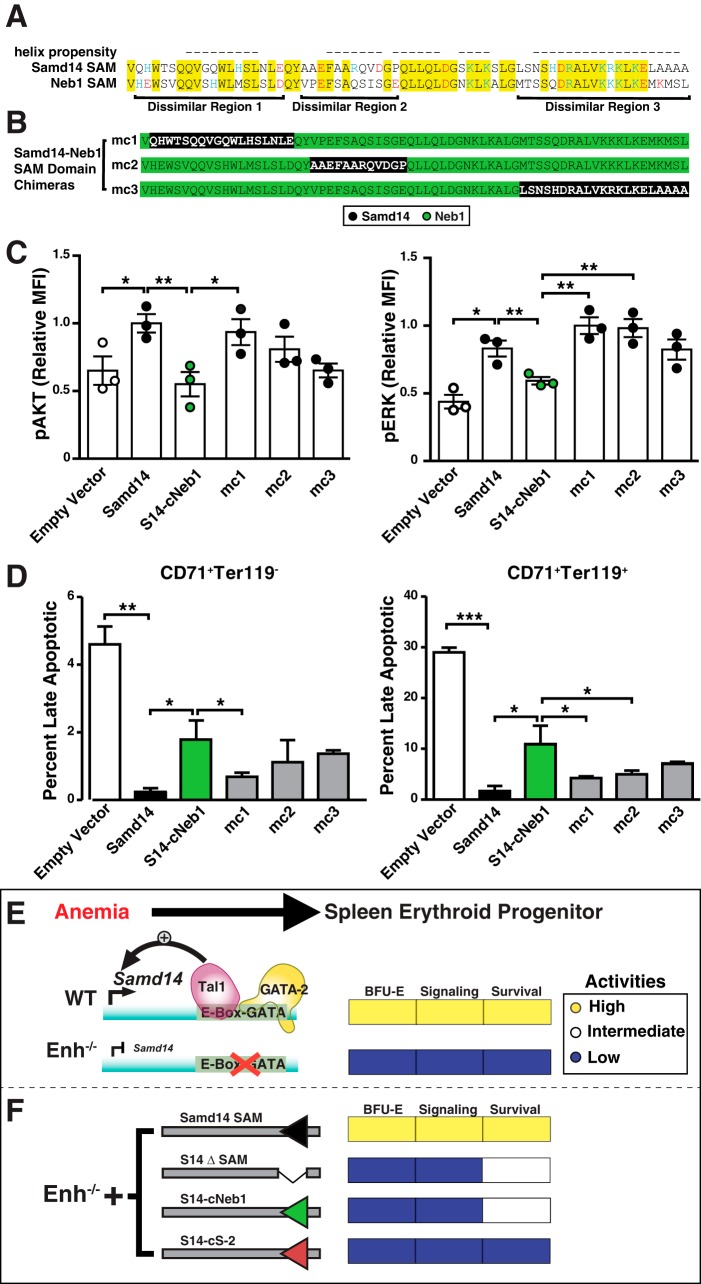
**Intrinsic differences in SAM domain activities.** *A,* alignment of Samd14 SAM (amino acids 323–390) and Neb1 (amino acids 986–1053) SAM domains. *Highlighted areas* indicate sequence identity. *Red* = negatively-charged amino acid. *Blue* = positively-charged amino acid. *B,* chimeric sequences of dissimilar regions of the Samd14 SAM domain (*black*) into the S14–Neb1 chimeric protein. *C,* quantitation of pAKT (*left*) and pERK1/2 relative (MFI) post-stimulation with 10 ng/ml SCF (*n* = 3 for each condition). *D,* quantitation of late apoptotic cells was segregated based on GFP^+^CD71^+^Ter119^−^ (*left*) and GFP^+^CD71^+^Ter119^+^ (*right*) followed by Draq7^−^AnnV^−^, live; Draq7^+^AnnV^+^. *Error bars* represent the standard error of the mean (S.E.). *, *p* < 0.05; **, *p* < 0.01 (two-tailed unpaired Student's *t* test). *E,* Samd14–Enh-activation in anemia promotes SCF/c-Kit signaling and regenerative erythropoiesis. In Samd14–Enh-knockout cells, Samd14 expression is lower, and SCF/c-Kit is reduced. *F,* rescue with full-length Samd14 restores signaling and erythroid progenitor function. The Samd14 sterile α-motif domain mediates Samd14 activities, including BFU-E colony formation, c-Kit-dependent MAPK/Akt signaling, and cellular survival. Although the SAM domains of Neb-1 and SHIP-2 are structurally-related to the Samd14 SAM domain, they lack the ability to promote signaling through SCF/c-Kit.

To delineate regions within the Samd14 and Neb1 SAM domains that functionally differ and sequences contributing to Samd14-specific functions, we aligned the Samd14 and Neurabin-1 SAM domains and identified three dissimilar sequences (Fig. 7*A*). Each unique region (termed mc1, mc2, or mc3) of the Samd14 SAM was tested for their ability to reconstitute activities of the Samd14–Neb1 chimera (Fig. 7*B*). Relative to S14–cNeb1-expressing cells, S14-mc1 exhibited 1.7-fold higher pAKT (*p* = 0.04) and pERK (*p* = 0.004) in response to SCF (Fig. 7*C*). S14-mc1 and S14-mc2 exhibited similar activity in pERK activation. Among GFP^+^CD71^+^Ter119^+^ and GFP^+^CD71^+^Ter119^−^, the percentage of late apoptotic cells was lower in S14-mc1 *versus* S14-Neb1 cells (Fig. 7*D*). These results demonstrate that the first and second α-helices of the Samd14 SAM domain uniquely contain sequences that regulate cellular signaling and survival in erythroid progenitors and are lacking in Neb1.

## Discussion

In response to anemia, one mechanism of stress resolution involves the GATA2-regulated activation of the Samd14–Enh intronic enhancer. This enhancer confers Samd14 expression and is required for survival of mice in severe anemia (4). Samd14 promotes c-Kit signaling *in vivo* and *ex vivo* (19). Dominant loss-of-function mutations in *Kit* or *SCF* exhibit a prominent hypersensitivity to chronic and acute anemia (7, 38–40). SCF-dependent dimerization of c-Kit activates multiple pathways that control hematopoietic development, homeostasis, cell survival, and proliferation, including AKT. Accordingly, SCF stimulates the expansion of *ex vivo*-cultured erythroid stress progenitors from mouse and human bone marrow (7, 41). Stress-dependent cellular responses during erythroid regeneration involve cooperation between SCF/c-Kit signaling and other signaling inputs, including Epo, BMP4, glucocorticoid receptors (42), and GDF15 (2, 7).

In this study, we innovated a genetic rescue assay to analyze Samd14 function in primary cells lacking the Samd14–Enh and established that Samd14 expression in mutant stress erythroid progenitors increases SCF/c-Kit signaling (Fig. 7*E*). Importantly, the rescue analysis provides rigorous evidence that the Samd14–Enh requirement for cell signaling and erythrocyte regeneration results from increased Samd14 expression and not from other mechanisms, *e.g.* regulation of genes in three-dimensional proximity that are not evident from linear chromosomal organization. Samd14–Enh is one of a cohort of GATA2 and anemia-activated enhancers predicted to regulate stress-dependent transcription in erythroid progenitors (4, 20). The Samd14–Enh requirement to control SCF/c-Kit signaling and stress erythropoiesis in mice expands the repertoire of activities of a canonical class of E-box–GATA enhancer elements beyond developmental and steady-state hematopoiesis to include regeneration (15, 20, 21).

Although SAM domains are predicted to share an equivalent secondary structure, their functional properties can differ in distinct contexts (24, 25). Single amino acid substitutions can drastically impact target binding affinity. For example, among a highly-related group of SAM domains, the ephrin cytoplasmic effector SHIP2 contains a SAM domain that binds the EphA2 SAM domain with high affinity, but does not interact with the EphA5 SAM domain (30). SAM domain-containing proteins are implicated in normal and pathologic mechanisms, including SAMHD1 in autoimmunity (27) and SAMD9/SAMD9L in myelodysplasia and myeloid malignancies (28). However, the role of the SAM domain in SAMD9/9L remains elusive (29, 43). Here, we describe an evolutionarily-conserved SAM domain within Samd14, which is highly expressed in hematopoietic stem and progenitor cells (HSPCs), myeloerythroid progenitors and progeny, and stress erythroid progenitors. The Samd14 SAM domain was required for Samd14 function to promote erythroid progenitor activity and c-Kit–mediated MAPK and AKT signaling. As the Samd14 SAM activities are not recapitulated with SHIP2 or Neb1 SAM domains, these studies revealed a SAM domain–specific function not predicted by apparent structural similarity (Fig. 7*F*). Samd14 SAM is therefore the founding member of the large SAM domain family with critical activities to control cellular signaling and survival.

Coding and noncoding *GATA2* mutations cause GATA2-deficiency syndromes, which often involve immunodeficiency and predisposition to develop myelodysplastic syndrome (MDS) and acute myeloid leukemia (AML) (15, 44–48). *Gata2* and *Samd14* transcription are both controlled by enhancers harboring a nearly-identical conserved E-box spacer–GATA composite element, and the study of these and related enhancers established a GATA2 and anemia-activated genetic network with many potentially-critical network constituents (2, 20). GATA2 strongly and directly activates Samd14 transcription, and Samd14 facilitates SCF/c-Kit signaling during hematopoiesis and survival of stress erythroid progenitors. Taken together with the importance of c-Kit signaling for normal and pathological hematopoiesis (18, 49), it is attractive to consider potential pathogenic consequences of Samd14 dysregulation. Our results establish a strong foundation to elucidate Samd14 mechanisms in nonmalignant and malignant hematologic pathologies and assess potential mechanistic similarities and differences between Samd14 and the MDS/AML-linked SAMD9/9L protein. The genetic rescue system described herein, which provided rigorous evidence for a unique Samd14 SAM function, will enable future studies to forge general principles governing this poorly-studied family of protein domains.

## Experimental procedures

### Plasmids

Hemagglutinin (HA)-tagged full-length Samd14 or Samd14 lacking the SAM domain (Samd14 ΔSAM) was cloned into mammalian expression plasmid pMSCV (Addgene catalog no. 21654) using BglII and EcoRI restriction digest sites. HA-tagged chimeric proteins in which coding sequences corresponding to 68-amino acid SAM domains of Neurabin-1 (from residues 986–1053) or SHIP-2 (from residues 1190–1257) replaced the 68-amino acid Samd14 SAM domain at the exact location, termed S14–cNeb1 and S14-cS-2, respectively. Smaller “micro-chimeras” were generated using the HA-tagged S14-Neb1 construct as a template to introduce 18-amino acid (mc1), 12-amino acid (mc2), or 21-amino acid (mc3) sequences from the corresponding Samd14 SAM domain (see Fig. 7*B* for sequences). Constructs were synthesized (Invitrogen and Twist Bioscience) and cloned into pMSCV using BglII and EcoRI restriction digest sites. All plasmids were retrovirally packaged with pCL-Eco (Addgene catalog no. 12371) in 293T cells.

### Mice and primary cell isolation

Samd14–Enh mutant mice were described previously (4). All animal experiments were carried out with ethical approval from the Association for the Assessment and Accreditation of Laboratory Animal Care at the University of Nebraska Medical Center. Hemolytic anemia was induced by a single dose of PHZ (100 mg/kg) (Sigma) administered subcutaneously. Spleens were harvested 3 days post-PHZ injection. Spleen and bone marrow from WT or Samd14–Enh^−/−^ mice were dissociated, resuspended in PBS with 2% fetal bovine serum (FBS), and passed through a 35-μm nylon filter to obtain single-cell suspensions. Erythroid precursors were isolated using negative selection lineage-depletion with biotin-conjugated antibodies and MojoSort streptavidin-conjugated magnetic nanobeads (Biolegend). Lineage-depletion antibody mixture included the following biotin-conjugated antibodies (Biolegend): anti-mouse CD3e (clone 145-2C11); anti-mouse CD11b (clone M1/70); anti-mouse CD19 (clone 6D5); anti-mouse CD45R (B220) (clone RA3-6B2); anti-mouse Gr-1 (clone RB6-C5); and anti-mouse Ter119. Following depletion, spleen erythroid progenitors were cultured in StemPro-34 media (Thermo Fisher Scientific) containing 2 mm l-glutamine, penicillin/streptomycin, 0.1 mm 1-thioglycerol, 1 μm dexamethasone, 0.5 units/ml erythropoietin, and 1% mSCF Chinese hamster ovary cell-conditioned medium and maintained at 2.5 × 10^5^–1 × 10^6^/ml. Bone marrow cells were cultured in Dulbecco's modified Eagle's medium containing 20% FBS, 4% IL-3–conditioned media, and 4% mSCF-conditioned media. For retroviral infections, 1 × 10^6^ lineage^−^ cells were added to 100 μl of viral supernatant, Polybrene (8 μg/ml), and HEPES buffer (10 μl/ml) and spinoculated at 1200 × *g* for 90 min at 30 °C. Cells were maintained at a density between 0.25 and 1 × 10^6^ cells/ml for 3 days. For cell-signaling assays, cells were serum-starved for 2 h in 1% BSA/IMDM at 37 °C and treated with 10 ng/ml SCF or vehicle for the indicated times. Cells were immediately fixed in 2% paraformaldehyde for 10 min at 37 °C and permeabilized in 95% methanol overnight at −20 °C.

### Flow cytometry

To obtain single-cell suspensions before antibody staining, cells were passed through a 35-μm nylon filter. All antibodies were from Biolegend unless otherwise stated. Lineage markers were stained with biotin-conjugated B220 (clone RA3-6B2), CD3ϵ (clone 145-2C11), CD11b (clone M1/70), CD19 (clone 6D5), Gr-1 (clone RB6–8C5), CD41 (clone MWReg30), CD16/CD32 (clone 93), CD34 (clone HM34), and TER-119 antibodies to enrich for BFU-E and CFU-E as described previously (36). APC–streptavidin (Biolegend) was used to detect all biotin-labeled lineage markers. Other surface proteins were detected with phycoerythrin (PE)-conjugated CD71 (R17217) and Fc receptor (12-0161); peridinin chlorophyll protein–Cy5.5-conjugated Sca1 (clone E13-161.7); APC-conjugated Ter119 (116212); and PE-Cy7–conjugated c-Kit (clone 2B8) (105814) antibodies. Analysis of erythroid maturation using CD71 and Ter119 was conducted as described (50). For intracellular antigens, methanol-fixed cells were stained with rabbit antibodies against phospho(Ser-473)-AKT (p-AKT) and phospho(Thr-202/Tyr-204) p44/42 ERK1/2 (p-ERK) (9271, 9101; Cell Signaling) for 30 min and then incubated in APC-conjugated goat anti-rabbit (1:200), PE–Cy7-conjugated c-Kit (1:200), and PE-conjugated CD71 (1:200) for 30 min at room temperature. Zombie UV (Biolegend) dye discriminated dead cells. Samples were analyzed using a BD LSR II or BD Fortessa flow cytometer. Values for pAKT and pERK levels were calculated by median fluorescence intensities (MFI) using FlowJo version 10.6.2 (BD Life Sciences) and normalized to the maximum overall value within each experiment (relative MFI). For applications requiring cell isolation, FACS was conducted on a FACSAria II (BD Life Sciences) and analyzed with FlowJo version 10.6.2.

### Western blotting

Proteins were boiled in SDS buffer (25 mm Tris, pH 6.8, 2% β-mercaptoethanol, 3% SDS, 5% bromphenol blue, 5% glycerol) for 10 min, resolved by SDS-PAGE, and detected with Pierce ECL Western blotting substrate (Thermo Fisher Scientific) on a LICOR imager. Antibodies used were polyclonal anti-Samd14 (4), anti-HA (Cell Signaling Technology), and anti-β-actin (Cell Signaling Technology). Secondary antibodies used are goat anti-mouse IgG–HRP and goat anti-rabbit IgG–HRP (Jackson ImmunoResearch).

### Immunofluorescence

Cells were cytospun at 2 × 10^5^ cells/ml of 50% FBS/PBS and fixed with 4% paraformaldehyde for 10 min at room temperature. Slides were washed with PBS, permeabilized with 0.2% Triton X-100 for 10 min at room temperature, and blocked with BlockAid Blocking solution (Invitrogen) for 1 h at room temperature in a humid chamber. Primary antibodies Samd14 (4), HA (Cell Signaling Technologies), and CD117/c-Kit (2B8; eBiosciences) were diluted in blocking solution and incubated overnight at 4 °C in a humid chamber. After washing with PBS-T (0.05% Tween 20), the slides were incubated with Alexa Fluor 594 donkey anti-rabbit (Invitrogen) or Alexa Fluor 647 goat anti-rat IgG (Invitrogen) for 1 h at room temperature, washed, and mounted with coverslip using Vectashield mounting media with DAPI (Vector Laboratories). Confocal images were acquired using the LSM800 (Carl Zeiss). All images were analyzed using Zen Software (Carl Zeiss).

### Structured illumination microscopy

Cells were serum-starved for 2 h in 1% BSA/IMDM at 37 °C and treated with 10 ng/ml SCF or vehicle. Cells were cytospun at 1 × 10^5^ cells/ml of 50% FBS/PBS, fixed with 4% paraformaldehyde for 10 min at room temperature, and immunostained with anti-HA (1:800) and anti-c-Kit (1:200) antibodies as described (see under “Immunofluorescence”). Structured illumination microscopy was performed using Carl Zeiss Elyra PS.1 microscope (magnification ×63; oil immersion lens). The images were analyzed in Zen software (Carl Zeiss), and ImageJ software was used to measure Pearson's co-localization coefficients (JaCoP plug-in) (51) in each cell (*n* = 4) (51).

### Colony-forming unit assays

CFU assays in PHZ-treated and retrovirally-infected spleen progenitors were conducted using a 0.3-ml spleen suspension at 1 × 10^6^ cells/ml (total spleen) or 3 × 10^5^ cells/ml (for retrovirally-infected GFP^+^ spleen) mixed with 3 ml of Epo, SCF, IL-3, and IL-6 Methocult M3434 (STEMCELL Technologies), and 1.1 ml was plated in replicate 35-mm dishes. Positive colonies were scored at 2 days (CFU-E) and 5 days (BFU-E) after plating.

### In silico modeling of SAM domains

The NMR structure of the Ship2 SAM domain was obtained from Protein Data Bank code 2k4p. The SAM domain sequences of human SAMD14 and Neurabin-1 (Neb1) were extracted from Uniprot as FASTA files and modeled using the YASARA homology modeling experiment (RRID:SCR_017591). The resulting structures were subjected to a 500-ps refinement simulation in pH 7.4 water at 298 K and 1 bar pressure. Na^+^ and Cl^−^ were used as counter ions to neutralize the simulation cell. The lowest energy structure from the refinement simulation for each molecule was selected for further analysis. RMSD of atomic positions between molecules was calculated in YASARA. To compute dipole moments (measured in debye (D) units), the Particle Mesh Ewald in a 10 Å simulation cell (YASARA) was conducted in vacuum using the Amber14 force field. Visual representation of charge is shown in the same orientation and automatically adjusted for optimal visualization.

### Statistics

For quantitation of cell number, cell colonies, Pearson's coefficient, and fluorescence intensities, the results are presented as means ± S.E. All analyses were conducted with GraphPad Prism. *p* values representing confidence in mean expression value differences were calculated using unpaired Student's *t* test with a cutoff of *p* < 0.05 deemed significant.

### Data availability

Formatted *in silico* projections of the Samd14 and Neurabin-1 SAM domains are available upon request (Kyle Hewitt, kyle.hewitt@unmc.edu). All other data are contained within the text.

## Author contributions

S. R., E. H. B., and K. J. H. conceptualization; S. R., L. C., and K. J. H. data curation; S. R., E. H. B., and K. J. H. formal analysis; S. R., E. H. B., and K. J. H. writing-review and editing; L. C., D. R. M., N. Y. P., and K. J. H. methodology; D. R. M. and N. Y. P. resources; N. Y. P. visualization; E. H. B. and K. J. H. funding acquisition; E. H. B. and K. J. H. investigation; K. J. H. writing-original draft; K. J. H. project administration.

## Supplementary Material

Supporting Information

## Footnotes

2 The abbreviations used are:

Epo: erythropoietin
SAM: sterile α-motif
SCF: stem cell factor
FBS: fetal bovine serum
HRP: horseradish peroxidase
MAPK: mitogen-activated protein kinase
ERK: extracellular signal-regulated kinase
MFI: median fluorescence intensity
CFU: colony-forming unit
CFU-GM: colony-forming unit–granulocyte/monocyte
CFU-E: colony-forming unit–erythroid
BFU-E: burst-forming unit–erythroid
EV: empty vector
Ann: annexin
DAPI: 4′,6-diamidino-2-phenylindole
MDS: myelodysplastic syndrome
AML: acute myeloid leukemia
IMDM: Iscove's modified Dulbecco's medium
PE: phycoerythrin
PHZ: phenylhydrazine
RMSD: root mean square deviation
EA: early apoptotic cell
LA: late apoptotic cell.
