# Supplementary material for: Sterile α-motif domain requirement for cellular signaling and survival
Source: J Biol Chem. 2020 Apr 2;295(20):7113–25. doi: 10.1074/jbc.RA119.011895 (PMC7242717; doi:10.1074/jbc.RA119.011895)
Supplement: Supporting Information [file supp_295_20_7113__index.html]

Sterile alpha motif domain requirement for cellular signaling and survival — Sterile Alpha Motif Domain Function — Sterile α-motif domain requirement for cellular signaling and survival — Sterile α-motif domain function — Supporting Information 

# Sterile α-motif domain requirement for cellular signaling and survival

## Supporting Information

- Supporting Information (to be published online) - This file contains Supplemental Table 1, Supplemental Figures 1-7 and Supplemental Figure legends
