# Supplementary material for: Sterile α-motif domain requirement for cellular signaling and survival
Source: J Biol Chem. 2020 Apr 2;295(20):7113–25. doi: 10.1074/jbc.RA119.011895 (PMC7242717; doi:10.1074/jbc.RA119.011895)

Supporting Information – Ray et al.

Figure Legends

Supplementary Table 1 – Percentages of GFP^+ cells (representing retrovirally-infected) in each experiment.

Supplementary Figure 1 - Representative flow cytometric histogram plots of pAKT and pERK staining after gating for $GFP^+Kit^+CD71^+$ retrovirally-infected cells. Related to data presented in Figure 1F.

Supplementary Figure 2 - Co-localization of Samd14 and c-Kit. (A) Representative structured illumination microscopy (SIM) images of spleen erythroid progenitors retrovirally-infected with HA-tagged Samd14 and a SAM domain-deleted mutant of Samd14 (S14 ΔSAM) stained with rabbit anti-HA antibody and AlexaFluor 594 (red), mouse anti-c-Kit antibody and AlexaFluor 647 (green), and DAPI (blue) in presence and absence of stem cell factor (-SCF/+SCF). 63X magnification, Scale bar = 2 μm (B) Pearson's correlation coefficient for Samd14 and c-Kit co-localization. (C) Pearson's correlation coefficient of Samd14 and DAPI co-localization. (D) Orthogonal projections of z-stack of spleen erythroid progenitors retrovirally-infected with HA-tagged Samd14 and S14 ΔSAM stained with antibodies for Samd14 (red), c-Kit (green), and DAPI (blue) in presence and absence of stem cell factor (-SCF/+SCF). 63X magnification, Scale bar = 2 μm. $*p < 0.05$ (two-tailed unpaired Student's t test).

Supplementary Figure 3 - Gating strategy for analysis of SCF-stimulated GFP^+Kit^+ cultures. (left) Representative flow cytometric analysis of GFP and forward scatter (FSC)

following retroviral infection in Samd14-Enh$^{-/-}$ spleen cultures. (right) Cells were subsequently gated for CD71 and c-Kit.

Supplementary Figure 4 - Samd14 or Samd14 Δ SAM Expression in Samd14-Enh$^{-/-}$ Bone Marrow. (A) Representative flow cytometric analysis of SCF-stimulated GFP^{+}Kit^{+} bone marrow cultures. (B) Quantitation of pAKT median fluorescence intensity (MFI) at 5 and 10 min post-stimulation with SCF (50 ng/mL) (N=3 in each condition). Error bars represent the standard error of the mean.

Supplementary Figure 5 - Dissecting Samd14 SAM Domain Activities in Cellular Survival with Chimeric SAM Domain Proteins. (A) Representative flow cytometric analysis of non-cell membrane permeating DNA dye (Draq7) and anti-Annexin V Pacific Blue (AnnV). Cells were first segregated based on GFP, CD71, Ter119 and c-Kit. Draq7^{-}AnnV^{-} = live, Draq7^{-}AnnV^{+} = early apoptotic (EA), and Draq7^{+}AnnV^{+} = late apoptotic (LA). (B) Quantitation of flow cytometric analysis of non-cell membrane permeating DNA dye (Draq7) and anti-Annexin V Pacific Blue (AnnV). Cells were first segregated based on GFP^{+}, CD71^{+}, Ter119^{-} and c-Kit^{+} (N=3). Error bars represent the standard error of the mean (SEM). ∗p < 0.05; ∗∗p < 0.01; ∗∗∗p < 0.001 (two-tailed unpaired Student's t test).

Supplementary Figure 6 - Localization of Samd14 chimeras. Representative immunofluorescence images of spleen erythroid progenitors retrovirally-infected with HA-tagged Samd14, Samd14 with SAM domain sequence of Neurabin-1 (S14-cNeb1) and Samd14 with SAM domain sequence of SHIP-2 (S14-cS2), stained with rabbit anti-HA antibody and AlexaFluor 594 (red) and DAPI (blue). 63X magnification, Scale bar = 10 μm.

Supplementary Figure 7 - Samd14 SAM domain increases the frequency of cells expressing BFU-E-associated surface markers in spleen progenitors. (A) Gating strategy and representative flow cytometric analysis of retrovirally-infected $Kit^{+}CD71^{low}$ and $Kit^{+}CD71^{low}$ in empty vector (EV), HA-tagged Samd14, a SAM domain-deleted mutant of Samd14 (Samd14 ΔSAM), Samd14 with SAM domain sequence of Neurabin-1 (S14-cNeb1) and Samd14 with SAM domain sequence of SHIP-2 (S14-cS2). FSC=forward scatter. (B) Left - Quantitation of the percent of retrovirally-infected $Kit^{+}CD71^{low}$ (N=3 in each condition). Right - Quantitation of the percent of retrovirally-infected $Kit^{+}CD71^{high}$ (right) (N=3 in each condition). * $p < 0.05$; **** $p < 0.0001$ (two-tailed unpaired Student's t test).

Supplementary Table 1

% GFP+ (related to Figure 1)

		N1	N2	N3	N4	N5	N6
**WT**	**EV 0 m**	30.3	28.6	27.1	13.3	30.3	28.6
	**EV 2 m**	33.7	26.4	21.7	25.2	33.7	26.4
	**EV 5 m**	40.6	27.3	26.2	24.8	40.6	27.3
	**EV 10 m**	44.5	34.7	31.6	19.5	44.5	34.7
	**EV 15 m**	19.9	25	24.6	28.4	19.9	25
	**EV 20 m**	17.5	17.8	26.1	21.9	17.5	17.8
**Enh-/-**	**Samd14 0 m**	20.7	20.5	38.9	16	20.7	20.5
	**Samd14 2 m**	20.9	19.5	17.6	22.8	20.9	19.5
	**Samd14 5 m**	38.7	51.6	24.3	25.5	38.7	51.6
	**Samd14 10 m**	34.3	44.3	36.2	59.2	34.3	44.3
	**Samd14 15 m**	29.3	42.2	42.3	55.4	29.3	42.2
	**Samd14 20 m**	24.2	43.6	32.8	43.1	24.2	43.6

% GFP+ (related to Figure 4C and D)

	N1	N2	N3
**EV**	27.8	33.8	32.8
**Samd14**	23.1	22.4	21.3
**delta SAM**	42.8	45.5	41.5

% GFP+ (related to Figure 5C, E, G, H)

	N1	N2	N3	N4
**EV 0 m**	23.6	32.4	20.4	49.2
**EV 2 m**	35.2	37	40.2	49.8
**EV 5 m**	41.8	28.7	41.2	50.9
**EV 10 m**	18.3	34.5	40	51.4
**Samd14 0 n**	5.87	16.1	30.9	40.4
**Samd14 2 n**	21.6	19.2	31.2	38.2
**Samd14 5 n**	18.3	22.7	29.1	33.1
**Samd14 10**	12.6	23.5	31.4	37.3
**deltaSAM 0**	42.8	42.1	45	18.1
**deltaSAM 2**	36.8	35.8	41.5	17.4
**deltaSAM 5**	39.7	25.9	41.5	18.6
**deltaSAM 1**	30.8	36.9	42.3	18.4

% GFP+ (related to Figure 6D-G)

	N1	N2	N3	N4
**Ev**	32.4	33.2	24.7	27.5
**Samd14**	27.5	33	29.5	34.5
**deltaSA**	29.8	32.2	30	30.7
**S14-cNe**	33.7	32.2	34.3	21.4
**S14-cS2**	32.6	24.8	33.3	22.8

% GFP+ (related to Figure 7C,D)

	N1	N2	N3
**Ev**	27.7	29	28.4
**Samd14**	31.8	31.8	31.9
**S14-cNeb1**	32.7	33.1	33.2
**S14-mc1**	30	31.5	31.3
**S14-mc2**	30.8	31.7	31.3
**S14-mc3**	40.5	39.8	40.5

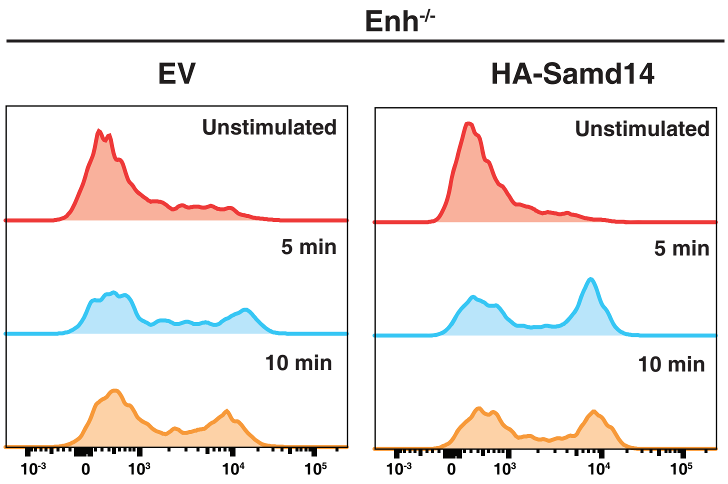
Enh-/-
EV
HA-Samd14
Unstimulated
5 min
10 min
Unstimulated
5 min
10 min
10-3
0
10³
10⁴
10⁵
10-3
0
10³
10⁴
10⁵

Supplementary Figure 2

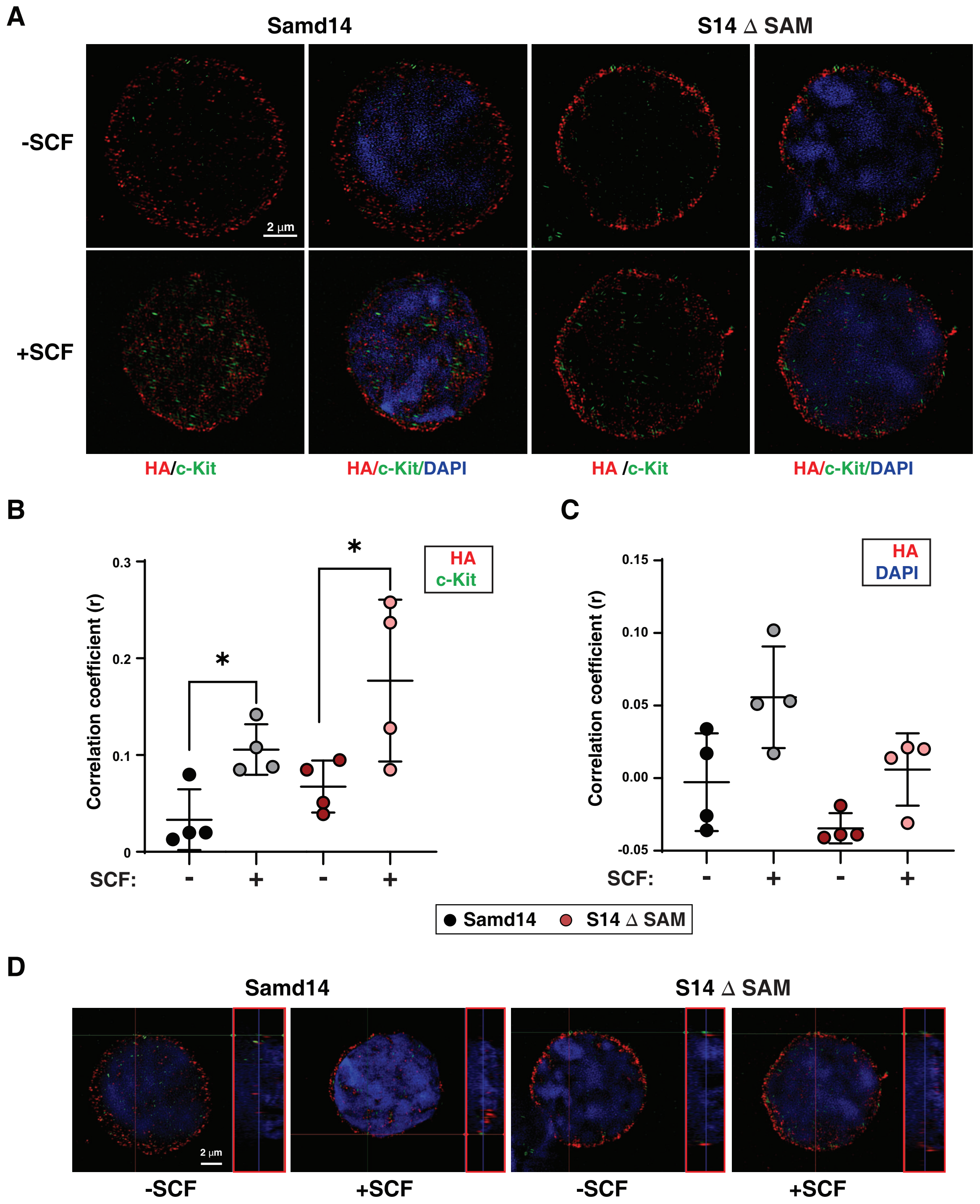

Supplementary Figure 3

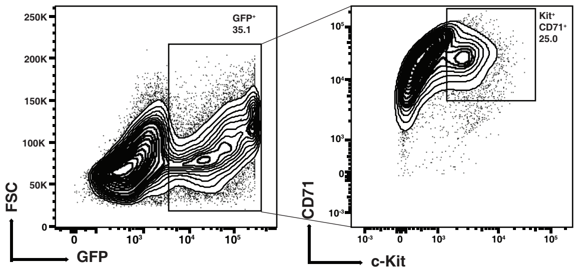

Supplementary Figure 4

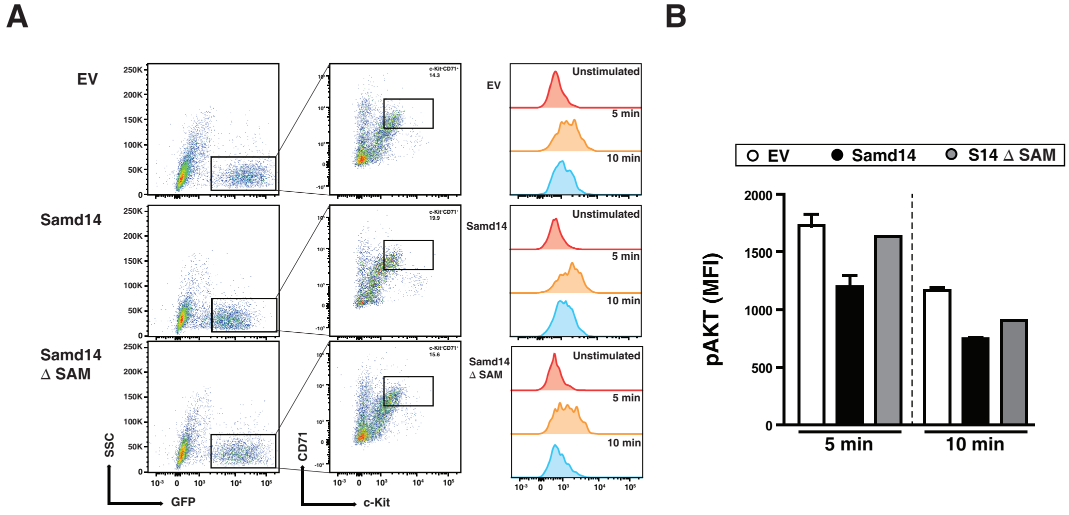

Supplementary Figure 5

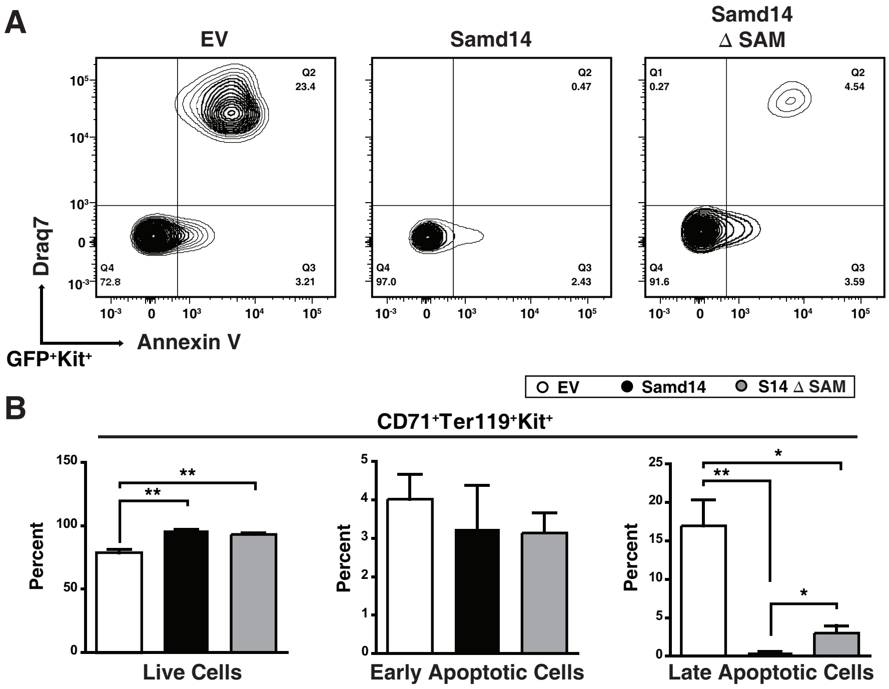

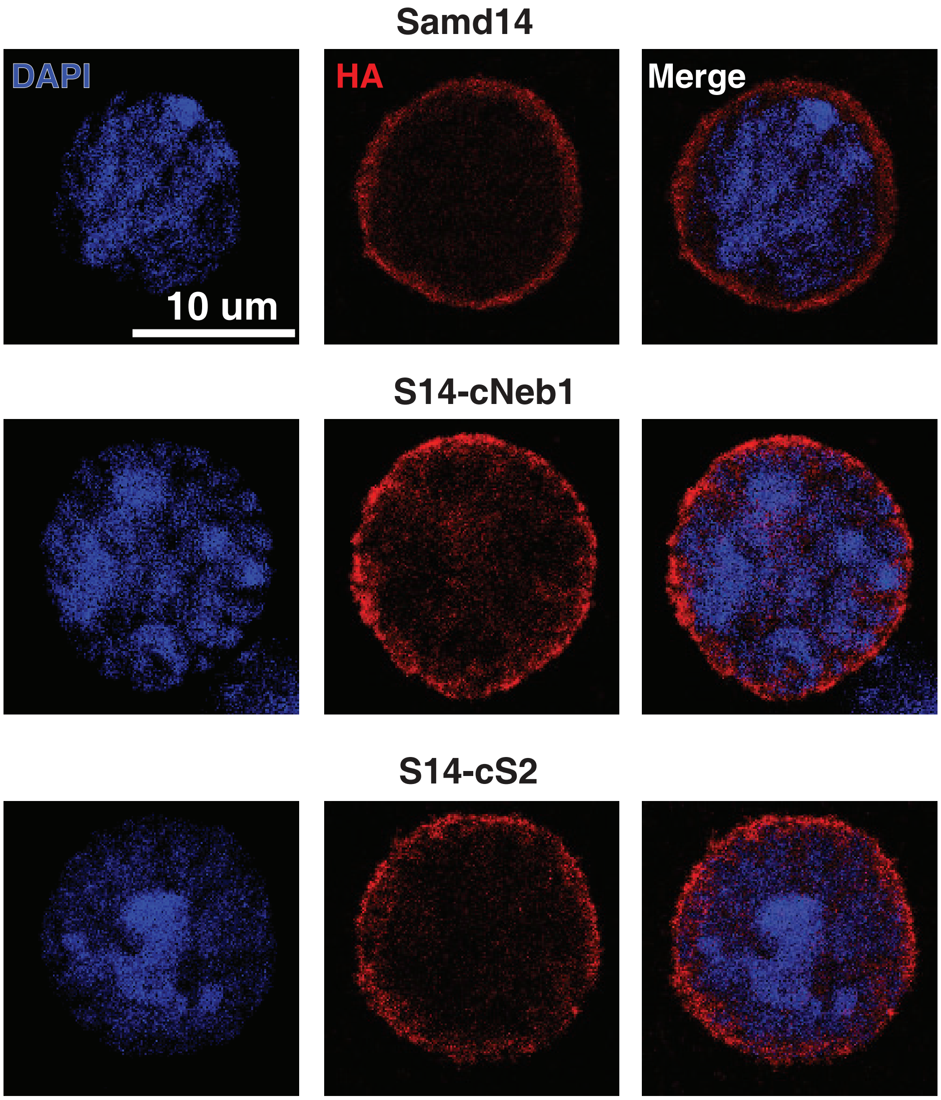
Samd14
DAPI
HA
Merge
10 um
S14-cNeb1
S14-cS2

Supplementary Figure 7

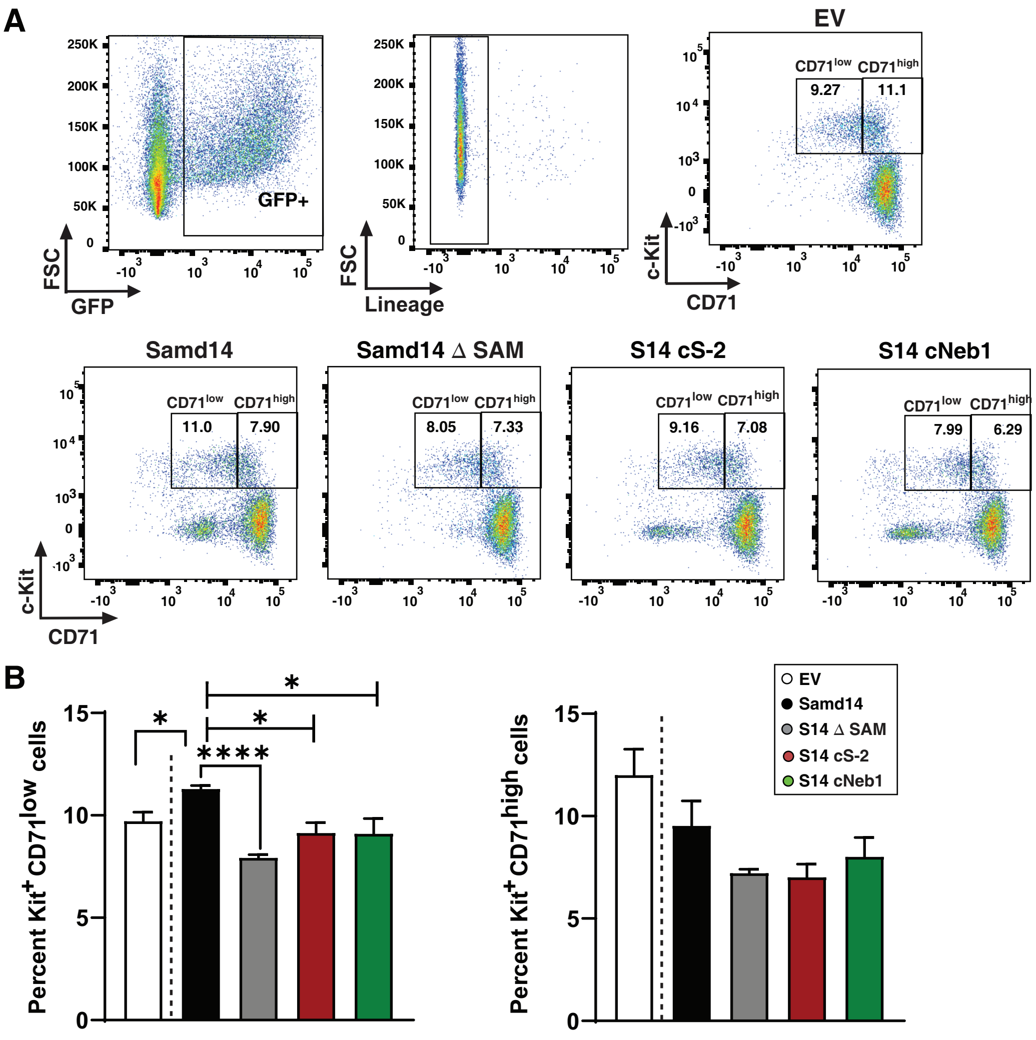

Supplement: Supporting Information [file supp_RA119.011895_157109_2_supp_503379_q83f55.pdf]
